# Diabetes in the hospitalized patients with urological diseases

**Published:** 2015

**Authors:** I Verde, E Rusu, E Suliman, A Costache, P Armean

**Affiliations:** * “Theodor Burghele” Hospital, Bucharest, Romania; ** “Carol Davila” University of Medicine and Pharmacy, Bucharest, Romania; *** ”N. Paulescu” Institute of Diabetes, Nutrition and Metabolic Diseases, Bucharest, Romania

**Keywords:** diabetes, urological diseases, hospitalization

## Abstract

**Background:** Diabetes and urological diseases are widespread health problems, whose incidence increases with age. The aim of this observational, retrospective study was to analyze the particularities of urinary disorders, which appeared in patients with diabetes, admitted in a urology ward.

**Material and methods:** A total of 6910 patients admitted in “Th. Burghele” Hospital from January 2013 to July 2014 were analyzed. Only admissions in urology wards and the first hospitalization of the patient were elements that were taken into account. Data was taken from the Hipocrate medical information system and Easy Medical Pro laboratory medical software. Study variables were age and sex of patients, the main discharge diagnosis, the number of days of hospitalization and the laboratory analyses collected on the day of admission. The data of the whole lot was analyzed and then an analysis on subgroups of patients was done.

**Results:** There were 16.52% (n=1142) patients with diabetes in the total group of analyzed patients. Urinary stones were the most frequent cause of hospitalization, both in patients with diabetes and in patients without diabetes (28.5%, respectively 37.5%). The average age was 60.01 years and the mean duration of hospitalization was 6.52 days. Patients with diabetes hospitalized for urinary stones, renal cancer, and infectious pathology were significantly older than patients without diabetes. The presence of diabetes prolonged hospitalization in the case of patients with kidney stones, kidney cancer and in those with infectious pathology. The most common malignancy was bladder cancer in both groups of patients. Malignancies were more common in diabetics (19.08% vs. 15.98%) and diabetes was a risk factor for malignancy in our study. In particular, patients with diabetes had a significantly increased risk of bladder cancer. In the analyzed group, diabetes was positively associated with prostate adenoma, genital infections, and prostate infections.

**Conclusions:** Diabetes increased the risk for certain urological diseases (bladder cancer, prostate adenoma, prostate and genital infections), it prolonged hospitalizations, and it was associated with certain features of laboratory analysis (leukocytosis, decreased glomerular filtration rate).

## Introduction

Diabetes and urological diseases are widespread health problems, whose incidence increases with age.

The most common urological complications occurring in patients with diabetes are bladder dysfunctions, sexual and erectile dysfunctions, and urinary tract infections [**1**]. These complications have a significant negative effect on the quality of life of men and women with diabetes.

Diabetes is a risk factor for the occurrence of kidney stones [**2**,**3**]. Nephrolithiasis occurs in at least 1 in 10 patients with diabetes [**3**]. 

Benign prostatic hyperplasia (BPH) is one of the most common benign tumors in men and occurs due to the irregular, non-malignant proliferation of the prostatic tissue. Although most of the literature supports the association between diabetes and BPH/ lower urinary tract symptoms (LUTS), many studies have failed to make a clear distinction between LUTS and BPH [**4**]. In patients with diabetes, both conditions may be favored, through complex, potentially disparate mechanisms, involving environmental, genetic, and hormonal factors, neuropathy and microvascular dysfunction.

Most studies to date have shown a positive association between diabetes and bladder cancer [**5**]. Diabetes causes a relative risk of bladder cancer 1.2-1.5 [**6**].

Morbidity and mortality caused by kidney cancer are increased in patients with diabetes comparative to general population [**7**]. Also, epidemiological studies have shown a negative association between prostate cancer and diabetes [**8**]. The urinary tract is the most common place for the infections in patients with diabetes [**9**]. Infections may be located in bladder (cystitis), in prostate (prostatitis), in kidneys (pyelonephritis) or in the genital area (orchitis, epididymitis).

## Objectives

The aim of this observational, retrospective study was to analyze the particularities of urinary disorders which appeared in patients with diabetes admitted in a urology ward.

## Material and Methods

A sample of 6910 patients admitted in “Th. Burghele” Hospital during a period of 18 months (from January 2013, until July 2014), were analyzed for various urological disorders. Only admissions in urology wards were taken into account. In case of multiple admissions, the first hospitalization of the patient was considered. The patients with diabetes were identified according to the diagnosis-related group (DRG) encode secondary diagnoses at discharge. Data was taken from the Hipocrate medical information system and Easy Medical Pro laboratory medical software. Study variables were age and sex of patients, the main discharge diagnosis, the number of days of hospitalization and the laboratory analyses collected on the day of admission (glycemia, blood count, creatinine and glomerular filtration rate (GFR) estimated by MDRD2, urinalysis and urine culture). Statistical analysis of data was performed by using SPSS version 17 software.

The data of the whole lot was analyzed and then an analysis on subgroups of patients was done - the patients with a discharge diagnosis of a confirmed malignancy (kidney, bladder or prostate cancer), the patients with prostate adenoma, the patients who had a discharge diagnosis of urinary lithiasis (nephrolithiasis, ureteral or bladder stones) and the patients hospitalized for infectious pathology (acute pyelonephritis, cystitis, acute prostatitis, orchitis or epididymitis).

## Results and discussion

There were 16.52% (n=1142) patients with diabetes and 83.47% (n=5768) patients without diabetes in the total group of analyzed patients.

**Fig. 1:**
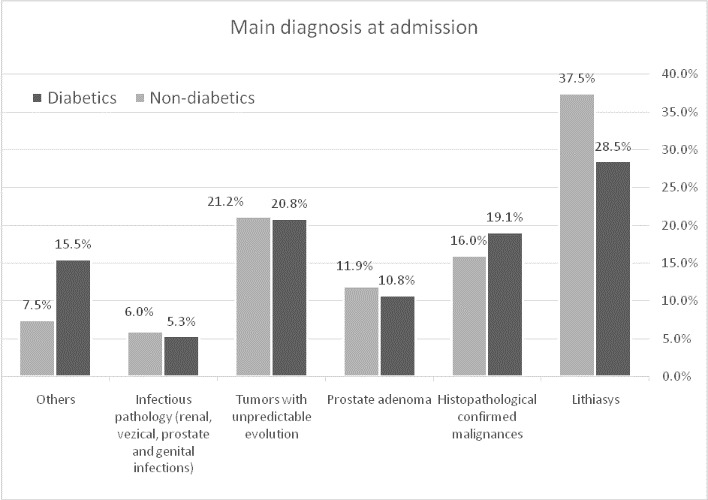
Main diagnosis at admission

Urinary stones were the most frequent cause of hospitalization, both in patients with diabetes and in patients without diabetes. Tumor pathology, histopathological unconfirmed, ranked second, followed by malignant tumor pathology, histopathologically confirmed (bladder cancer, prostate cancer and renal cancer). Prostate adenoma was the fourth cause of hospitalization, both in diabetic and in nondiabetic patients. Then came the infectious pathology (genital infections, prostate infections, bladder and kidney infections) (**Fig. 1**).

**Fig. 2:**
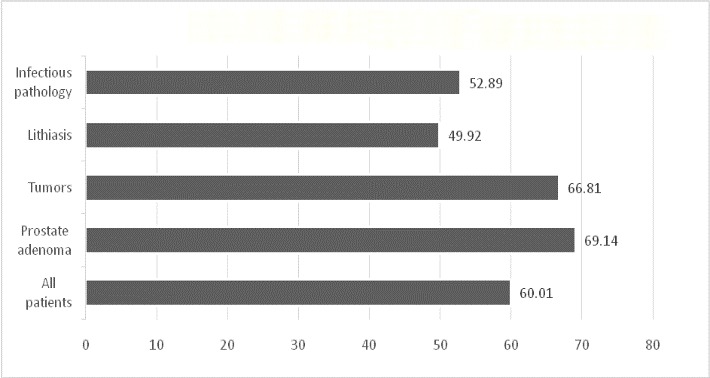
Age at time of admission

The average age of the lot was 60.01 years. Compared with the rest of the patients, the patients hospitalized for prostate adenoma and tumor pathology (confirmed or unconfirmed histopathologically) were significantly older (p=0.000 <0.001) and those hospitalized for infectious pathology and lithiasis were significantly younger (p=0.000 <0.001). The age at time of admission, for various pathologies is represented in (**Fig. 2**).

**Fig. 3:**
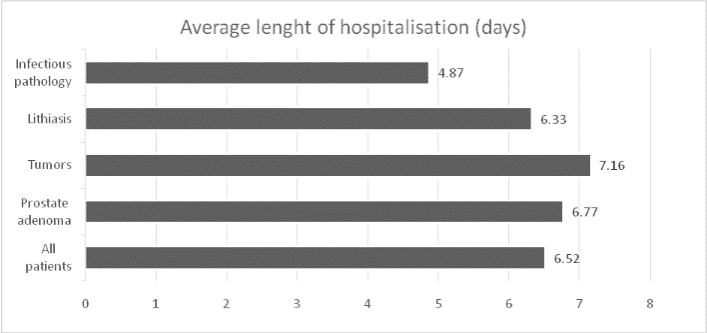
Duration of hospitalization

The mean duration of hospitalization, for the whole lot, was 6.52 days. Compared with the rest of the patients, patients admitted for tumor pathology (confirmed or unconfirmed histopathologically) had significantly more days of hospitalization (7.16 days, p=0.000 <0.001), and those admitted for infectious pathology and lithiasis had significantly less days of hospitalization (6.33 days and 4.87 days, p=0.000 <0.001).The days of hospitalization, for various pathologies are described in (**Fig. 3**).

**Urinary stones**

**Fig. 4:**
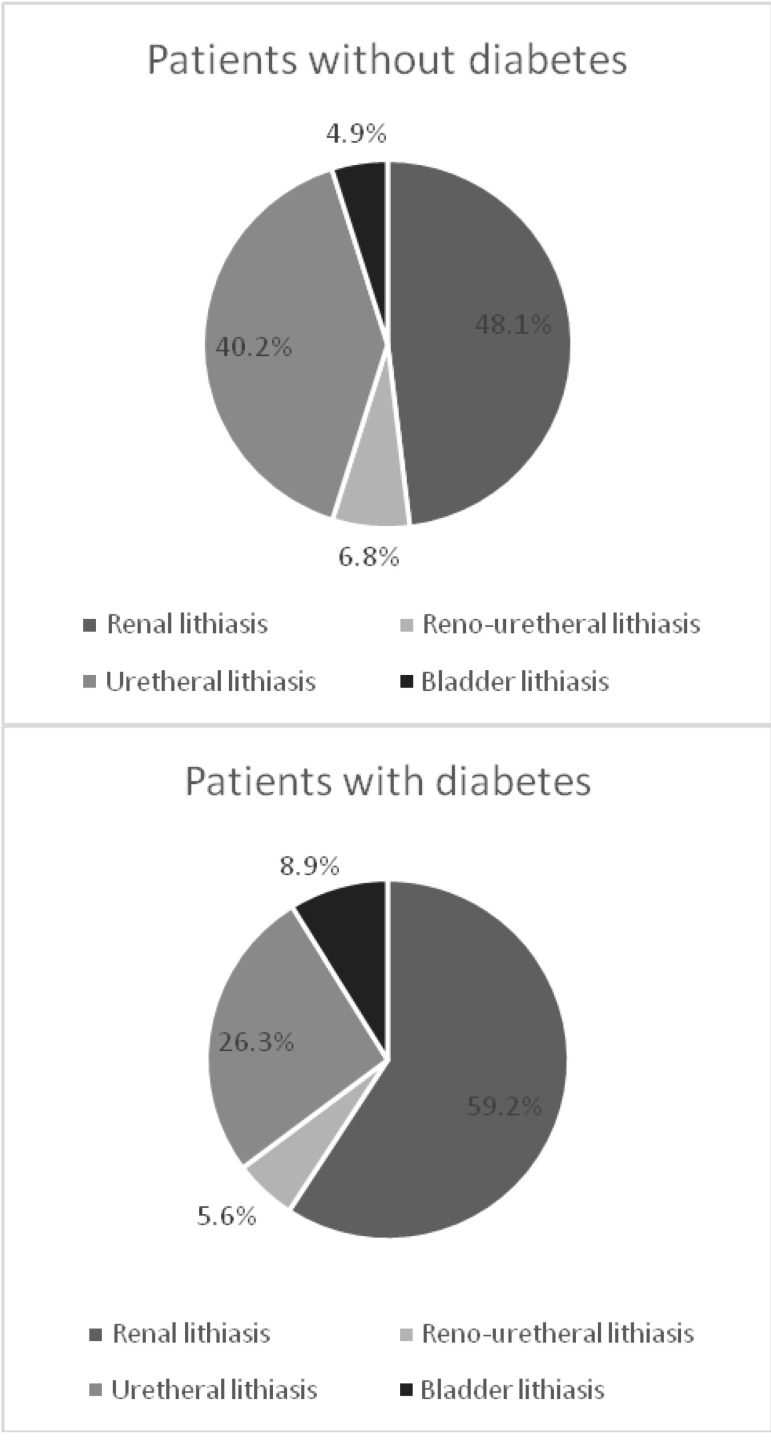
Urinary lithiasis

Patients with lithiasic pathology (kidney, ureteral or bladder stones) were 33.99% (n=2487), from whom those with diabetes were 13.1% (n=325). The distribution of diabetic and nondiabetic patients with urinary lithiasis according to the location of the stones is represented in (**Fig. 4**).

Among diabetic patients with kidney and ureteral stones, the percentages of women and men were approximately equal (49.8% women and 50.2% men), whereas in patients without diabetes, women were more frequently than men (55.1% versus 44.9%), but the difference between the two groups was not statistically significant. Patients with bladder stones were most frequently men in the both groups (90.9% in the group of diabetic patients and 83.9% in nondiabetic patients). Patients with diabetes and kidney or ureteral stones were statistically significantly older than those without diabetes (the mean age at admission was 60.09 years in diabetic patients and 47.63 years in nondiabetic patients) (p<0.05) and they required a longer period of hospitalization than the nondiabetic patients (6.95 days compared with 6.28 days) (p<0.05).

The glucose level was positively correlated with age at the time of admission, with creatinine and white blood cells (WBC). There was no statistically significant linear correlation between blood glucose on admission and the length of stay. But, in the group of patients who had the blood glucose lower than 180 mg/ dl at admission, the average days of hospitalization were 4.87, while in the group of patients who had the blood glucose higher than 180 mg/ dl at admission, the average days of hospitalization were 6.47, and the difference was statistically significant (p<0.05).

There was no statistically significant difference between the frequency of urinary infection at admission between diabetic and nondiabetic. Both diabetic and nondiabetic patients who had leukocytosis at admission, had a longer duration of hospitalization, but the difference was statistically significant only in the group of patients without diabetes (6.37 days vs. 5.36 days, p<0.05). Diabetic patients with urinary stones had an estimated glomerular filtration rate (after MDRD2) less than nondiabetic patients and the difference was statistically significant (61.7 ml/min/m2, respectively 72.7 ml/min/m2, p <0.05).

**Confirmed malignant pathology**

Patients with malignant disease were 16.49% (n=1140), from whom those with diabetes were 19.1% (n=218).

**Fig. 5:**
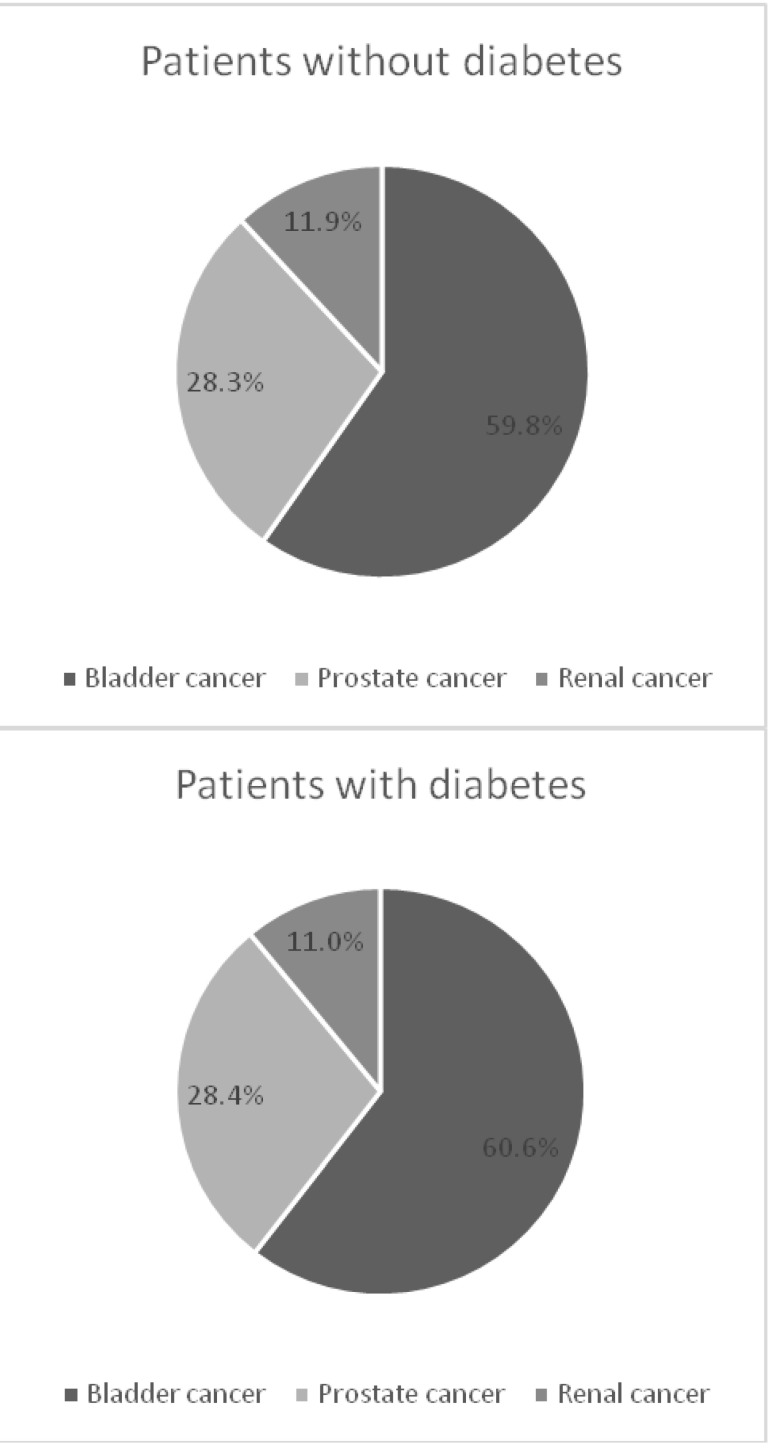
Malignancies histologically confirmed

The most common malignancy was bladder cancer - 59.91% (59.76% in patients without diabetes and 60.55% in patients with diabetes), followed by prostate cancer - 28.33% (28.30% in diabetics and 28.44% in nondiabetics) and renal cell carcinoma - 11.75% (11.93% to 11.00% in patients with diabetes and in nondiabetic patients) (**Fig. 5**).

The average blood glucose on admission in the group of diabetic patients with prostate cancer was 158 mg/ dl, in the group diabetic patients with bladder cancer was 151.5 mg/ dl, and in the group of diabetics with renal cell cancer was 163.4 mg/ dl. Differences between the mean blood glucose at admission on different pathologies were not statistically significant and the glucose level in diabetic patients group was not positively correlated with length of stay.

In the group of diabetic patients with bladder tumor there were 82.6% males (n=109) and in that of nondiabetics 77% males (n=424). The average age at admission in diabetic patients was lower than in nondiabetic patients (65.9 years vs. 67.02 years), but the difference was not statistically significant. Patients with diabetes and bladder tumor which had leukocytosis at admission (WBC >10,800/ mm3) had significantly more days of hospitalization than those without leukocytosis (8.5 days vs. 5.5 days) (p <0.05).

In the subgroup of patients with prostate cancer and diabetes, the average age at admission was higher than in the subgroup of nondiabetic patients with prostate cancer (68.88 years vs. 67.93 years), but the difference was not statistically significant. Patients with diabetes and prostate cancer who had a urinary infection at admission, had a longer hospital stay than those without a urinary infection (5.5 days vs. 3.7 days) (p<0.05). There was no statistically significant difference between the mean prostate-specific antigen (PSA) at admission in diabetic patients with prostate cancer as compared to nondiabetic patients with prostate cancer (21.7 ng/ ml vs. 22.9 ng/ ml, p = 0.866).

There were 70.8% males (n=17) in the subgroup of patients with diabetes and renal cancer and 58.2% males (n=64) in nondiabetic patients with kidney cancer. The average age of diabetics was significantly greater than that of patients without diabetes (68.2 years vs. 60.8 years) (p<0.05). In the case of patients with diabetes and renal cancer, hospitalization lasted significantly longer than in the case of nondiabetic patients with renal cancer (14.5 days vs. 10.6 days) (p<0.05).

Malignancies (kidney cancer, bladder and prostate cancer) were more common in diabetics (19.08% vs. 15.98%) and diabetes was a risk factor for malignancy in our study, with odds ratio [OR] =1.24 (95% confidence interval [CI] 1.05-1.46) (p<0.05). Also, patients with diabetes had a significantly increased risk of bladder cancer, with OR=1.23 (95%CI 1.01-1.51) (p<0.05).

**Prostate adenoma**

There were 12.2% (n=843) patients with prostate adenoma in the whole group, from whom those with diabetes mellitus were 22.7% (n=191). Diabetes was a risk factor for prostate adenoma in the analyzed group, with OR=1.576 (95%CI 1.322-1.878) (p<0.05). The average blood glucose on admission, among patients with diabetes and prostate adenoma, was 158.17 mg/ dl. Blood glucose levels on admission did not significantly correlate with the days of hospitalization. The average age at admission (69 years in the group with diabetes and 69.19 years in the group without diabetes) and number of days of hospitalization (6.76 days in diabetics and 6.77 days in nondiabetic) were approximately equal in both groups. Patients with diabetes and prostate adenoma had an average higher WBC on admission and the difference between groups (diabetics and nondiabetics) was statistically significant (p=0.015<0.05). There were no statistically significant differences between diabetics and nondiabetics in the value of eGFR at admission (70.8 ml/min/m2 in diabetics, respectively 74.12 ml/min/m2 for nondiabetics) and in the value of PSA (6.24 ng/ ml for diabetics and 6.10 ng/ ml for nondiabetics).

**Infectious pathology**

Patients hospitalized for infectious pathology accounted for 5.8% of total admissions (n=401), from whom patients with diabetes were 14% (n=56).

The average age at the time of admission was significantly higher among patients with diabetes (63.43 years for diabetics, respectively 51.17 years for nondiabetics) (p=0.000<0.001) and the hospitalization period was significantly higher for the diabetics group compared to patients without diabetes (5.96 days, respectively 4.69 days) (p=0.008<0.05). The average blood glucose on admission in the group of patients with diabetes was 174.9 mg/ dl. Patients with diabetes had an average number of WBC on admission higher than those without diabetes (10798 WBC/ mm3 in diabetics compared to 8307 WBC/ mm3 for nondiabetics) and a lower eGFR (62.82 ml/min/m2 in diabetics, respectively 77.20 ml/min/m2 for nondiabetics), and the difference was statistically significant (p=0.000 <0.001).

**Fig. 6:**
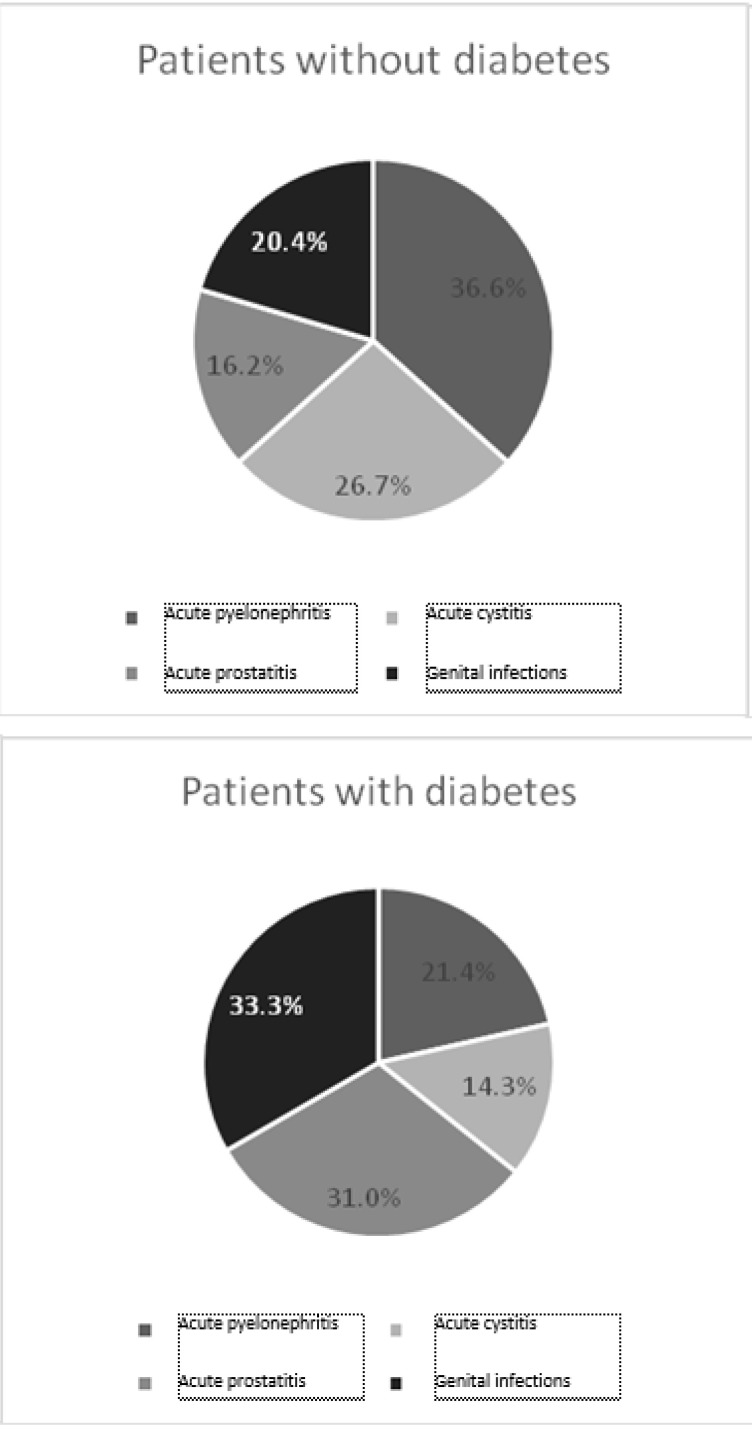
Acute infectious pathology

The distribution of patients with diabetes and without diabetes, depending on the different locations of the infection is shown in (**Fig. 6**).

Acute tubulointerstitial nephritis was the main diagnosis at admission in 1.1% (n=79) of the patients, from whom those with diabetes were 11.4% (n=9). Acute cystitis was the cause of hospitalization for 0.8% of the patients (n=57), from whom diabetic patients were 10.5% (n=6).

Genital infections (orchitis, epididymitis, orhiepididymitis with or without abscess) were present in 0.8% of the patients (n=53), from whom those with diabetes were 26.4% (n=14). Diabetes was a risk factor for infectious pathology of the genital area in the analyzed group with OR=1.606 (CI95% 1.021-2.525).

Patients with prostate infections (acute prostatitis) were 0.6% of total group (n=44), from whom those with diabetes were 29.5% (n=13). Diabetes was a risk factor for acute prostatitis in the analyzed group, with OR = 1.606 (95%CI 1.112-4.085). There were no statistically significant differences in the average value of PSA between diabetic and nondiabetic hospitalized patients with acute prostatitis (2.99 ng/ ml in diabetics, respectively 3.88 ng/ ml for nondiabetic, p=0.522).

## Conclusions

Urinary stones were the most frequent cause of hospitalization in both groups of patients (with or without diabetes).

In the group of analyzed patients, diabetes was a risk factor for malignant diseases and in particular for bladder cancer. Also, diabetes mellitus was positively associated with prostate adenoma and prostate and genital infections.

The presence of diabetes was associated with the prolongation of hospitalization in patients with kidney stones, kidney cancer and in those with infectious pathology.

There were no statistically significant differences between the frequencies of various type of cancers in the group of diabetic patients compared to those without diabetes.

Diabetic patients requiring hospitalization for various urological pathologies frequently had leukocytosis and an estimated glomerular filtration rate lower than nondiabetic patients.

Blood glucose at admission was not positively correlated with the length of stay for the most studied urological disorders.

In the case of prostate pathology (prostate adenoma, acute prostatitis and prostate adenocarcinoma), there were no significant differences in terms of PSA value between diabetics and nondiabetics.
